# Report of Cases of Extragenital Chancre

**Published:** 1898-02

**Authors:** Burnside Foster

**Affiliations:** St. Paul


### REPORT OF CASES OF EXTRAGENITAL CHANCRE.

BY BURNSIDE FOSTER, M. D., ST. PAUL.

The following cases are reported, without comment, as a contribution to the literature of extragenital syphilis, the importance of which has been so markedly emphasized and so much more generally recognized since the publication of Buckley's valuable monograph, "Syphilis Insontium." I have notes of several other similar cases, but as there existed in each of them a reasonable doubt as to the diagnosis, I have thought it best to report only those which were fully substantiated.

### CHANCRE OF LIP.

Miss K., age 19. First consulted me August 7, 1890. For about three weeks has been troubled with an obstinate sore on the inside of the lower lip. Examination showed the lower lip to be swollen to about twice its normal size, and at about the center of the right half the mucous membrane was the seat of a lesion about the size of a dime, very slightly excavated at its centre and surrounded by a considerable area of redness. The mass was indurated, thick and slightly painful on pressure. There was not much secretion from the ulcerated surface but it bled quite easily. The cervical glands were enlarged on both sides. The patient denied any sexual intercourse, but admitted that she had been very free with her lips with various men, and had permitted almost every other liberty with her person, so that there had been ample opportunity for infection. About three weeks later a general eruption followed by numerous mucous patches and later by alopecia established the diagnosis.

### CHANCRE OF RIGHT FOREFINGER.

Dr. E. consulted me first August 15, 1893, for an obstinate ulcerating condition of the right forefinger. About two weeks previously he had first noticed a sore point at the base of the nail which soon became red and ulcerated and caused the entire finger to swell. There had been very little discharge, but a tough grey slough had formed in the bottom of the sore and it was quite painful. On examination the end of the finger was swollen to almost twice its natural size; there was a deep excavated lesion about the size of a dime, filled with necrotic tissue and surrounded by a red induration. The nail was lifted from its bed and there were unhealthly looking granulations beneath it. There was distinct enlargement of the epitrochlear and axillary glands, which were not painful. The patient complained of headache and said he had been chilly with slight fever for the last two nights. I told him that the sore was, in my opinion, a chancre and directed him to use a hot bichloride wash three times daily, and dress the sore with a calomel and iodoform ointment. He repudiated the diagnosis but adopted the treatment. About five weeks later he wrote me that the sore was nearly healed but that his hair was falling out, and that he was covered with an eruption which he did not describe. I afterwards learned that he went to Chicago where the diagnosis of syphilis was confirmed and a course of treatment instituted.

CHANCRE OF TONGUE.

Mrs. R. G., age 32, an attractive widow of good social position in a neighboring town, was referred to me by her family physician, March 20, 1894, for an obstinate ulceration of the under side of the tongue near the tip. She was engaged to be married at the time to a man whom I had seen some months before in consultation, and who had numerous patches in his mouth. The lesion was undoubtedly a chancre and her physician afterwards wrote me that she soon developed a typical syphilis with severe constitutional symptoms.

CHANCRE OF RIGHT FOREFINGER.

Dr. S., first seen June 15, 1895. About eight weeks ago first noticed a soreness about the nail of finger on outer side. After treating it for some time with antiseptic washes and mild ointments without any effect, it was vigorously cauterized with fuming nitric acid. This treatment only made it worse, and for a time there was a deep ulceration which involved most of the end of the finger. The specific nature of the sore was not suspected until about ten days previous to his first consultation with me, when he broke out with a general pustular eruption, accompanied by headache, pain in bones and slight fever. On examination the chancre is still unhealed and, besides the eruption, there was general adenopathy, especially of axillary glands, and several mucous patches in mouth, with a dusky red condition of the mucous membrane of the tonsils and pharynx. Prompt specific treatment was instituted and the patient has been under observation for about two years and is now apparently well. In this case the source of infection could not be ascertained, as the patient had been doing a large obstetric and gynæcological practice.

CHANCRE OF LEFT NIPPLE.

Miss K. R., age 24, a prostitute, consulted me January 4, 1896, for a sore nipple. She said she had frequently allowed men with whom she consorted to kiss the nipple. About six weeks previously a man had bitten it, not severely, but so that for a day or two afterwards it was a little sore. About two weeks before I saw her the nipple had become quite sore and inflamed. On examination the base of the nipple and the skin adjoining is the seat of an indurated ulceration about the size of a chestnut, surrounded by a considerable area of redness; no other smyptoms. She was given a bichloride wash and an emollient ointment and told to report in a week. January 20 she returned with a general papular syphilodeum, enlarged axillary and cervical glands, headache, pain in limbs and slight fever. She was at once placed upon specific treatment and is still under occasional observation, although she has been very irregular in taking her medicine, and still has numerous patches and sore throat.

### CHANCRE OF RIGHT FOREFINGER.

Dr. K., first seen April 20, 1897. On January 10, 1897, he attended a woman in confinement, whose husband was known to have syphilis. February 28, noticed a rather painful sore in right forefinger. At first this appeared to be an infected scratch and little attention was paid to it, although it refused to heal. Early in April the patient began to feel ill and feverish, and about two weeks previous to consulting me noticed a general macular eruption over body. On examination the remains of the original sore appeared as a somewhat diffused redness of the finger, slightly tender but not ulcerated. There was a general papular syphilide with marked enlargement of the axillary and cervical glands. The patient has since developed sores in mouth and throat, which readily yielded to specific treatment. When last seen he was doing well, and he is at present on a vigorous course of mercury.

### CHANCRE OF GUM.

Miss A. B., age 18. Seen in consultation May 28, 1897. In December, 1896, patient had considerable trouble with one of the right upper molar teeth and had considerable work done by a dentist. The tooth was filled with soft filling and the trouble subsided. About four weeks later the gum became sore and swollen and there developed an ulceration which would not heal. Early in February, about six weeks after the sore first appeared, the patient broke out with a general eruption, accompanied by headache and some fever, which was supposed to be measles, although there was no coryza or eye symptoms. This was followed by alopecia and sores in the mouth and on the tongue. The eruption gradually faded and disappeared in about a month. When seen in May the patient was pretty well covered with a pustular eruption, on the body and scalp, which had existed for a couple of weeks. There was general adenopathy, the glands in the neck being especially prominent. A few mucous patches existed in the mouth and around the genitals. There was redness and a marked induration of the gum at the site of the original sore.

It seemed probable that the dentist's instrument introduced the infection, but there was of course the possibility that it had come from some other contact.

CHANCRE OF TONSIL.

Miss E., age 27. First seen July 28, 1897. This patient was referred to me by Dr. J. H. Stewart, who had been treating her for iritis, of which he suspected the specific origin. Some six weeks ago she had a severe "ulcerated sore throat." On examination there is now a deep ulceration of the right tonsil, which is much swollen, and there is a general diffuse redness of the throat. The glands on the right side of her neck are considerably enlarged and there exists on the body and face a general papular eruption, which is somewhat scant. The patient has improved very markedly under mercury and is still under treatment. The method of infection could not be traced.—*Northwestern Lancet.*

